# Eleven quick tips for running an interdisciplinary short course for new graduate students

**DOI:** 10.1371/journal.pcbi.1006039

**Published:** 2018-03-29

**Authors:** Timothy E. Saunders, Cynthia Y. He, Patrice Koehl, L. L. Sharon Ong, Peter T. C. So

**Affiliations:** 1 Department of Biological Sciences, National University of Singapore, Singapore; 2 Mechanobiology Institute, National University of Singapore, Singapore; 3 Department of Computer Science and Genome Center, University of California Davis, Davis, California, United States of America; 4 Singapore MIT Alliance for Research and Technology Centre, Singapore; 5 Department of Biological Engineering, Massachusetts Institute of Technology, Cambridge, United States of America; Genome Quebec, CANADA

## Abstract

Quantitative reasoning and techniques are increasingly ubiquitous across the life sciences. However, new graduate researchers with a biology background are often not equipped with the skills that are required to utilize such techniques correctly and efficiently. In parallel, there are increasing numbers of engineers, mathematicians, and physical scientists interested in studying problems in biology with only basic knowledge of this field. Students from such varied backgrounds can struggle to engage proactively together to tackle problems in biology. There is therefore a need to establish bridges between those disciplines. It is our proposal that the beginning of graduate school is the appropriate time to initiate those bridges through an interdisciplinary short course. We have instigated an intensive 10-day course that brought together new graduate students in the life sciences from across departments within the National University of Singapore. The course aimed at introducing biological problems as well as some of the quantitative approaches commonly used when tackling those problems. We have run the course for three years with over 100 students attending. Building on this experience, we share 11 quick tips on how to run such an effective, interdisciplinary short course for new graduate students in the biosciences.

This is a *PLOS Computational Biology* Education paper.

## Introduction

With dramatic advances in biological approaches, experimental techniques, and computational analyses, many modern researchers in cell biology require an interdisciplinary background. These researchers need strong training in both biology and quantitative reasoning [1]. However, few (if any) undergraduate courses equip students with the necessary breadth of tools (e.g., molecular biology, biochemistry, cell biology, bioinformatics, statistics, coding, image analysis, data handling, and algorithmic thinking) that are needed to undertake many graduate research studies [2], not to mention the theories behind those tools. Furthermore, biologists and physical scientists are trained differently, with different emphases on mathematical and quantitative approaches. Hence, it can be challenging for them to communicate clearly about the same problems [3,4]. An example is the concept of biological precision [5], which biologists, physicists, and engineers approach from different perspectives. As a consequence, we have found that students gravitate toward laboratories that align with their previous experience, thereby reducing the potential for interdisciplinary collaborations. Furthermore, students are often reluctant to try new techniques and ideas that fall outside of their areas of expertise.

As an attempt to address these challenging issues, we (following the examples of others [6–8]) have instigated an interdisciplinary short course specifically aimed at new graduate students. The overall objectives of the short course are to: (i) develop interdisciplinary skills and reasoning early on in a graduate student’s career; (ii) ensure that our incoming students have a common foundation in biology and quantitative skills; and (iii) encourage teamwork and collaboration amongst graduate students. The course involves an intensive 10 days of (morning) lectures and (afternoon) hands-on experience before students begin their graduate studies and research. The course has grown from 19 students in 2015 to 60 in 2017. Most of the students come from across Asia, though each year we have students from Europe and Australasia. Morning lectures range from fundamentals of cell biology to optics and statistical analysis. The afternoon sessions focus on developing programming skills (utilizing primarily MATLAB, as well as R and Python) and encouraging interdisciplinary teamwork.

### Eleven quick tips for a successful interdisciplinary course

Having run the course for three years, we have gained critical experience and developed ideas about how to effectively engage students with interdisciplinary methods in biology and encourage them to move outside their comfort zones. Below, we outline 11 tips for instigating a successful interdisciplinary short course for new graduate students.

1. *Clear objectives*. The course is aimed at new graduate students; those students will have come from a broad range of backgrounds in terms of the topics they studied as undergraduate students, as well as in terms of the culture of teaching they were exposed to. Therefore, the course aims and objectives need to be clear from the beginning to set student expectations. We provide precourse reading material, which consists of papers that exemplify the crossover between biology and the physical sciences [9–14]. We also provide the course content and objectives via a website (https://mbi.nus.edu.sg/event/2017-bootcamp-on-mechanobiology/).
  We conduct a precourse survey for which students are given a small mark to incentivize completion. This allows us to test whether student expectations are aligned with the envisaged course objectives and also makes sure the course is focused on student needs. Furthermore, it enables us to better understand the quantitative background of the incoming students. The first session of the short course should be devoted to going clearly through the course objectives and highlighting points of interest from the questionnaire. By introducing not just the objectives of the course, but also the rationale for implementing such objectives, we help the students feel that they are part of the process instead of just consumers of the course. We implemented this survey in 2017 and saw a marked improvement in student perception of the course compared to previous years. In particular, students felt that the course was better aligned to their needs.
  An important note is that the students should appreciate that the course will not make them experts in interdisciplinary research or in a particular area [15]. To gain deeper understanding requires participation in longer courses, and we regularly highlight such suitable courses through the program. One of our principal aims is to help students identify their own weak areas and guide them on how to improve.
2. *Quality over quantity*. The students have a wide variety of backgrounds, from cell biology to materials engineering. They will be conducting a wide variety of biological research, from ecology to tissue engineering. Clearly, there are no courses that can satisfactorily cover all the topics and methods that they really need to know in 10 days. We strongly believe that it is desirable to resist the temptation to include the full spectrum of those topics. Instead, the course should focus on core topics that are representative of local research focus and strength and directly relevant to the students’ needs. The intent is to illustrate the relationships between biology and quantitative sciences with well-chosen examples, not to superficially cover all possible topics. We recommend choosing a single theme per morning of lectures. We typically use three lecturers per morning to cover that theme (e.g., structure of the cell), who each bring a different perspective.
  The practical sessions should directly build on the content from the lectures. For example, image analysis immediately follows morning lectures on biological imaging. We find that using the lecture material helps to frame the problems for students and encourages engagement with the course material. Furthermore, we deliberately use techniques that cannot be performed in spreadsheets. For example, we spend an afternoon exploring clustering of biological data using a coding environment (e.g., MATLAB, R, Python)—something that cannot be implemented straightforwardly in software environments such as Excel—yet this is an approach that is invaluable in fields as diverse as ecology [16] and cancer biology [17].
3. *Breadth*, *not depth*. From our experience, one of the most challenging parts of forming an effective course is getting the balance between providing enough content such that the students are exposed to a range of ideas and not overwhelming them. We have found that the best solution is to focus on exposing students to a core set of new ideas and approaches particularly aimed at quantitative biology. Often, students are able to fill in gaps in their knowledge through discussions with their peers [18]. Depth of understanding can and will be achieved by additional, more in-depth graduate classes.
  Getting the balance between providing breadth and not overburdening students is challenging. We have carefully refined our course over the past three years in response to feedback from both the students and the faculty teaching the different lectures and practicals. We currently have 12 hours of lectures on core biology, 12 hours of lectures on quantitative methods, and >25 hours of practicals. The latter includes both laboratory and computational work.
4. *Student and lecturer engagement*. The schedule should include informal time periods where participants can engage with lecturers outside of the formal lecture hours. Including such time periods encourages students to proactively engage with the lecturers, i.e., to move beyond a general undergraduate attitude that does not encourage (and sometimes even discourages) such interactions. Indeed, we ask lecturers to actively engage and interact with students. This includes developing a more active lecture environment in which students have to participate, as well as getting the lecturers to be present and participate in coffee and lunch breaks. Another key point is to make sure that the lecturers and class assistants are not only aware of but also supporters of the idea of increased student–instructor interactions. We note that several lecturers have used internet-based tools to get real-time feedback and interact with students. Such initiatives have greatly enhanced student participation and in-class learning.
5. *Timing of the course*. We run the course during the two weeks that precede the start of the fall semester. We negotiate with the students’ host departments to ensure minimal conflicts between the course schedule and the specific departmental requirements for new students (such as safety briefings). Finally, the transition from undergraduate study to graduate research is major; by running the course at the beginning of their studies, we help students identify gaps in their knowledge and also set expectations for how hard students must work to achieve a high-quality graduate experience.
6. *Group work*. Have strong group work components that are clear from the beginning [19]. A major aim is to enhance student communication both with faculty and each other. We create groups with a mixture of scientific backgrounds: every group (each with four students) has at least one scientist from a STEM (science, technology, engineering, mathematics) discipline and one biologist. Amongst the biologists, we try to make sure that there is diversity within each group (e.g., medics, plant biologists). All coursework is designed to foster interdisciplinary discussion. Assessment should also include rewards for effective group work. We have a single report compiled per group with a common mark or grade. Each report is centered around a topical issue in biology, such as issues in quantifying fluorescent images, the risks associated with the Zika virus, and CRISPR-Cas9 editing. The report topics are given early on in the course to allow the students time to discuss amongst themselves and also with lecturers about the material. We also provide a tutorial at the end of the course dedicated to the report writing, in which students are provided with working space and lecturers are available for feedback and discussion. Most students give positive feedback about this form of assessment. In previous years, we gave the students a week between the end of the course and submission of the essay. However, we found that some students did not contribute after lectures finished. Consequently, in 2017, we allowed only a three-day gap between the end of lectures and submission of the written report. This timeline was agreed upon through negotiation with the students and reflected the demands on their time from other courses.
7. *Quantitative skills*. Teaching quantitative skills should be central to the planning and is best conducted through hands-on experience. Directly linking these quantitative skills—such as extracting information from an image—to the course material is essential. As the range of backgrounds of our students are very varied, we do not grade the quantitative work. Instead, marks are awarded for engagement and effort. Students are encouraged to help and teach each other with coding and practical sessions. Indeed, we encouraged students to form groups—typically of similar coding ability—to work through the problem sets. We provided notes, but other than a 15-minute introduction to each session, no lectures were given. Students were encouraged to “get their hands dirty” as soon as possible. Qualitatively, this approach appears to be enhancing the breadth of skills used by students during their doctoral studies, with supervisors reporting an increased willingness amongst students to incorporate quantitative skills within their work, in particular in microscopy data analysis.
8. *Relaxed atmosphere*. The course is aimed at building interactions and knocking down barriers between disciplines. Therefore, creating a relaxed atmosphere is essential. We run the course more like a workshop, where there is a program book (allowing easy note taking), name tags, and plenty of breaks (including free coffee). Such an environment is more conducive for interdisciplinary discussions. To avoid groups splintering, try to make sure that rooms for lectures and practicals are nearby to maximize interactions. Furthermore, the schedule should be intensive to ferment strong interactions but also include social events—collaborations are as often built over a drink as they are over the bench. We had three evening social events, where students bonded outside of class. We also include an outing for faculty and students to explore parts of Singapore.
9. *Dynamic feedback*. Use online surveys for interactive teaching and feedback. Each year, the cohort of students will have different demands, so the course needs to be flexible. An example here is the decision on how much time should be allowed before the students turn in their written report after the end of lectures, as this time may be constrained by other commitments. We found that the students responded positively to being asked to give feedback throughout the course and to help us focus the content and teaching on their needs. After the rigidness of many undergraduate courses, this approach further encourages the new graduate students to think more independently and to shape their own learning.
10. *Reward effort*, *and no exam*. Grades should be focused around participation, interaction, and engagement. A key element is that relative improvement is measured in assessing coding and practical work. Many of our students come from undergraduate courses that focused on passing exams rather than building scientific knowledge, and encouraging students to break away from the rigidity of such systems is essential. Unsurprisingly, many students like the lack of an exam. To grade student performance, we marked their participation and engagement with the course. In particular, at the end of every hands-on session, the lecturers and teaching assistants met to go through marks for students, highlighting those who had excelled or underperformed. Proactivity during the course—such as asking questions about underlying concepts and working constructively with peers—is specifically rewarded.
11. *Connectedness*. The course we describe here has been carefully aligned to our needs at the National University of Singapore. In adapting such a course for your own cohort, remember to maintain connectedness in the material. The relevance of tools and skills that students learn during the course should be easily demonstrable. For example, through the course, students (i) prepare samples, (ii) image the samples, and (iii) perform image analysis on their own data, before finally (iv) creating a figure demonstrating their results. This figure also included a detailed legend with interpretation of the results. Students further prepared a report on a hot topic in biology (see also tip 6), which required integration of concepts from both biological and physical sciences to achieve high grades.

## Discussion

When discussing this course with fellow academics, we are often asked how the course appeals to students with such a broad range of backgrounds. First, for the biologists, the content is heavily focused on the cell cytoskeleton and its mechanical properties (due to the Mechanobiology Institute at the National University of Singapore). In particular, many cell processes of general interest are represented in a novel manner, focusing on physical interactions and forces rather than genes. Therefore, although a number of topics are sometimes repetitive in content, the focus and philosophy of the teaching are different. For students from the physical sciences, some have a good grounding in the general principles of optics. However, few undergraduate courses go into detail on identifying the optics that are suitable for imaging biological specimens, e.g., confocal microscopy. It is also rare to have undergraduate courses that cover fluorescence in depth. Over the past two years, we have had very few complaints of the course content not being relevant or interesting.

We have found that the short course we describe here appears to be effective for our incoming students in developing their interdisciplinarity, but that has come with experience. After each running of the course, it is essential to critically assess what worked and what did not. We are proactive at gaining student feedback, with over 70 feedback reports over the past three years. Further feedback from lecturers and the student academic supervisors is garnered, with these suggesting that students are more willing to engage with a broader range of ideas and to use quantitative techniques. Informal interviews with students suggest that students are using more quantitative approaches in their work. Overall, it is critical to have student buy-in for such an interdisciplinary course at the interface of the physical and biological sciences to be effective. This is achieved primarily by embedding material and ideas from the physical sciences directly relevant to biological research within the course.

As a note of caution, short courses can potentially give a false impression of learning: while both students and faculty report high satisfaction with such courses (partly due to the limited time commitment), recent work has questioned whether students are truly learning in such programs [15]. The focus of our course is not to displace more in-depth longer courses (including year-long interdisciplinary programs [4]), which have been shown to be more effective in teaching detailed ideas [20]. Instead, our course is focused on developing the breadth of student thinking and engagement through: (i) exposing students to new ideas and directions; (ii) developing their interdisciplinary awareness and “language” skills; and (iii) growing their teamwork skills. Qualitative assessment of student performance in laboratory rotations and from discussion with supervisors suggests that we are meeting these aims. Of course, the real test of this is whether the work of the students is more interdisciplinary. This can be assessed through analysis of students over the next few years as the first batch of students who attended the course will finish their graduate studies.
